# Supplementary material for: Management of ocular adverse reactions to antiglaucoma medications–Survey of optometrists in Ghana
Source: PLOS Glob Public Health. 2025 Oct 9;5(10):e0004251. doi: 10.1371/journal.pgph.0004251 (PMC12510571; doi:10.1371/journal.pgph.0004251)
Supplement: Attachment — Submitted filename: PLUS JOURNAL COMMENTS.docx [file pgph.0004251.s002.docx]

1. In line 75, it should be ‘’there’’ instead of ‘’here’’ after According to Kyei, Koffuor (11)
2. SPSS has changed its name to Statistical Product and Service Solutions instead of Statistical Package for the Social Sciences
3. In line 121 and 122, evaluating relationships between explanatory and response 122 variables and maintaining significance at p ≤ 0.05 is an incomplete statement . What statistical method of analysis was used in the evaluation
4. Explain how the ethical principles of anonymity, beneficence, malficence and Justice were applied in this study.
5. In line 327, it should be ---our findings are consistent and NOT our findings is consistent
6. What makes lack of further training and awareness of reporting systems for OADRs is a widespread problem. Substantiate this assertion with evidences
7. The research implications in this study are week. Research implications are the consequences of research findings. They go beyond results. Research implication should inform further research, shape policy, or spark new solutions to old problems.  Research Implications should link to specific results within your research to ensure they’re grounded in reality. Research implications need to be rational and based on data from your research, not conjecture. An evidence-based approach to implications will lend credibility and validity to your work.
