# Supplementary material for: Management of ocular adverse reactions to antiglaucoma medications–Survey of optometrists in Ghana
Source: PLOS Glob Public Health. 2025 Oct 9;5(10):e0004251. doi: 10.1371/journal.pgph.0004251 (PMC12510571; doi:10.1371/journal.pgph.0004251)
Supplement: Attachment — Submitted filename: Response to Reviewers.docx [file pgph.0004251.s004.docx]

**`Reviewer#1**

1. **Comment:** In line 75, it should be ‘’there’’ instead of ‘’here’’ after According to Kyei, Koffuor (11).

**Response:** Thank you for noting this. We have revised the text to replace "here" with "there" in line 75 to ensure proper context and clarity in the sentence.

2. **Comment:** SPSS has changed its name to Statistical Product and Service Solutions instead of Statistical Package for the Social Sciences.

**Response:** We have updated the manuscript to reflect the correct name, "Statistical Product and Service Solutions," in place of "Statistical Package for the Social Sciences."

3. **Comment:** In line 121 and 122, evaluating relationships between explanatory and response 122 variables and maintaining significance at p ≤ 0.05 is an incomplete statement. What statistical method of analysis was used in the evaluation?

**Response:** Thank you for pointing this out. The analysis of the relationships between the explanatory and response variables was conducted using inferential statistical methods. Specifically, we employed chi-square tests. This method was used to determine the statistical significance of the relationships, with significance levels set at p ≤ 0.05. We have updated the manuscript to include this clarification in lines 121–122.

4. **Comment:** Explain how the ethical principles of anonymity, beneficence, maleficence and Justice were applied in this study.

**Response:** Thank you for your valuable question. Below is an explanation of how these ethical principles were applied in this study:

i. **Anonymity**: Participants' anonymity was ensured by assigning code numbers to all collected data, with no personal identifiers recorded. The consent form explicitly informed participants that their responses would remain confidential and could not be linked to them in any way. Additionally, no names or identifiable information were included in publications or reports stemming from the study.
ii. **Beneficence**: The study was designed to contribute valuable insights into optometric care practices, aiming to improve patient outcomes and professional development among clinicians. Efforts were made to minimize any inconvenience to participants by ensuring the questionnaire was concise and the participation process straightforward.
iii. **Non-Maleficence**: To prevent harm, participants were informed that they could withdraw from the study at any time without providing an explanation, and they could skip any questions they found uncomfortable or private. The research procedures were non-invasive, and there were no physical or psychological risks to participants.
iv. **Justice**: The principle of justice was upheld by ensuring fairness in participant selection. All optometrists within the defined inclusion criteria were given an equal opportunity to participate, without discrimination. This approach ensured that the study's benefits were distributed equitably across the target population.

In addition to these principles, the study adhered to the Declaration of Helsinki, with ethical approval obtained from the Committee on Human Research, Publication, and Ethics at Kwame Nkrumah University of Science and Technology, Kumasi, Ghana (CHRPE/AP/1038/23). Informed consent was sought from all participants before their involvement. We have included these clarifications in the revised ethical considerations section of the manuscript.

5. **Comment:** In line 327, it should be ---our findings are consistent and NOT our findings is consistent

**Response:** Thank you for identifying this grammatical oversight. The sentence has been corrected to read "our findings are consistent" in line 327.

6. **Comment:** What makes lack of further training and awareness of reporting systems for OADRs is a widespread problem. Substantiate this assertion with evidence.

**Response:** Thank you for your question. The lack of further training and awareness of reporting systems for ocular adverse drug reactions (OADRs) is a widespread issue in Ghana due to several factors. This was captured in the problem statement of the background section.

First, adverse drug reactions (ADRs), including those related to antiglaucoma medications, are a significant concern, as they can lead to serious consequences such as eye irritation, redness, blurred vision, and iris pigmentation (Bhatt et al., 2005; Detry-Morel, 2006). Despite this, there is a gap in further training for optometrists and other eye care professionals on how to recognize and properly report these reactions.

. A study by Kyei et al. (2014), showed that while patients reported adverse effects from medications such as alphagan, timolol, and xalatan, there is limited awareness and systematic reporting among optometrists in Ghana. The same study by Kyei et al. (2014) found that although adverse reactions were documented, the lack of effective reporting structures or awareness of such systems among healthcare providers, including optometrists, hinders the systematic collection and analysis of these reactions.

The increasing prevalence of glaucoma in Ghana, which is the leading cause of irreversible blindness (Resnikoff et al., 2004), highlights the need for better awareness of the potential ADRs associated with antiglaucoma medications. Without effective reporting systems, many ADRs go unreported, hindering the opportunity for improved patient care and drug safety monitoring. Addressing this issue requires enhanced training for optometrists on identifying and reporting OADRs and implementing accessible reporting systems to better track and manage these reactions.

7. **Comment:** The research implications in this study are weak. Research implications are the consequences of research findings. They go beyond results. Research implication should inform further research, shape policy, or spark new solutions to old problems.  Research Implications should link to specific results within your research to ensure they’re grounded in reality. Research implications need to be rational and based on data from your research, not conjecture. An evidence-based approach to implications will lend credibility and validity to your work.

**Response:** Thank you for your insightful comment. We appreciate the opportunity to improve the implications and recommendations section. Upon reviewing the feedback, we have revised the section to ensure that the implications are more directly grounded in the data and findings of the study.

In the original version, we discussed the importance of training programs and standardized reporting systems for OADRs, drawing on similar findings from other studies. However, we acknowledge that the implications should be more explicitly connected to our specific results.

In response to your comment, we have revised the implications to link them to our findings, ensuring they are grounded in the research results. For example, our study identified significant gaps in optometrists' awareness and training regarding the management and reporting of OADRs. Based on this, we now explicitly state that the implementation of targeted, evidence-based training programs, grounded in the challenges identified in our data, will enhance the ability of optometrists to effectively recognize and report adverse drug reactions. Additionally, we emphasize the need for policy recommendations around the establishment of clear, accessible reporting systems, drawing directly from the challenges identified in the study.

We have also incorporated more specific recommendations for future research, such as exploring the effectiveness of training programs and the impact of standardized reporting systems on the documentation of OADRs in Ghanaian eye care. This ensures the implications are not only evidence-based but also actionable and linked directly to the study’s findings.

We hope these revisions address the concerns raised and provide a more solid foundation for the research implications and recommendations.

**Reviewer#2**

**Comment 1:** *As per the title of the manuscript "MANAGEMENT OF OCULAR ADVERSE REACTIONS TO ANTIGLAUCOMA MEDICATIONS – SURVEY OF OPTOMETRISTS IN GHANA" here the management is missing in the manuscript so it is not at all justifying the title.*

**Response:** Thank you for pointing this out. The manuscript discusses the management strategies employed by optometrists for OADRs, including advising patients on medication changes, reducing exposure to preservatives, and providing symptomatic relief. To ensure clarity and alignment with the title, we have refined the discussion to explicitly highlight these management practices and their implications.

**Comment 2:** *The adverse reaction symptoms data are collected from the optometrist, not from the patient directly, it may create bias.*

**Response:** We also acknowledge the limitation inherent in collecting data from optometrists rather than directly from patients and this was stated in the limitation section. However, this approach aligns with the study’s objective to assess optometrists’ perspectives, knowledge, and practices regarding OADRs. Optometrists are the clinicians who routinely assess and record patient–reported symptoms as part of the clinical management process. Data collected by these clinicians are often reliable and consistent as they are trained to recognize and document symptoms experienced by patients. Hence relying on patients to report adverse drug reactions may introduce more recall bias especially when the symptoms may have occurred in the past and or are subtle.

**Comment 3:** *Semi-structured questionnaires are not attached, and any validation by an authority person is missing.*

**Response:** Thank you for this observation. We have now included the semi-structured questionnaire used in the study as a supplementary file to the manuscript. Additionally, the questionnaire was well-reviewed by the ethical clearance committee.

**Comment 4:** *p-value is not significant.*

**Response:** We acknowledge the reviewer's concern regarding the lack of statistical significance for some results. While not all p-values in our study met the conventional threshold for significance (p ≤ 0.05), the observed trends and patterns in the data offer valuable insights into the management of ocular adverse drug reactions (OADRs). The findings, even in cases where statistical significance was not achieved, contribute to understanding the broader context of optometric practices and pharmacovigilance in Ghana. These non-significant results should not be dismissed but can be interpreted as part of a nuanced narrative. Emphases were placed on significant values that provides a balanced perspective that underscores both statistically significant findings and meaningful trends that may inform future research and practice.
