# Supplementary material for: Management of ocular adverse reactions to antiglaucoma medications–Survey of optometrists in Ghana
Source: PLOS Glob Public Health. 2025 Oct 9;5(10):e0004251. doi: 10.1371/journal.pgph.0004251 (PMC12510571; doi:10.1371/journal.pgph.0004251)
Supplement: Attachment — Submitted filename: Response to Reviewers (1).docx [file pgph.0004251.s005.docx]

Title**: MANAGEMENT OF OCULAR ADVERSE REACTIONS TO ANTIGLAUCOMA MEDICATIONS – SURVEY OF OPTOMETRISTS IN GHANA**

Response to reviewers’ comments

**Reviewer#3**

1. **Comment:** Add a brief description of how the questionnaire was pre-tested (e.g., pilot testing with a sample group, expert review by ophthalmologists/statisticians) in the Methods section

**Response:** Thank you for your insightful suggestion. We have revised the methods section to include a brief description of how the questionnaire was pre-tested. The questionnaire was pilot tested on eight licensed optometrists, and based on their feedback, it was revised to improve clarity, relevance, and ease of understanding prior to its final administration.
