# Supplementary material for: Management of ocular adverse reactions to antiglaucoma medications–Survey of optometrists in Ghana
Source: PLOS Glob Public Health. 2025 Oct 9;5(10):e0004251. doi: 10.1371/journal.pgph.0004251 (PMC12510571; doi:10.1371/journal.pgph.0004251)
Supplement: Attachment — Submitted filename: Response to Reviewer R2.docx [file pgph.0004251.s006.docx]

**Response to Reviewer’s comment**

***Comment #5: Is the manuscript present in an intelligible fashion and written in standard English?***

*Reviewer #4 comment:* No

***Response to Reviewer #4****:* We thank Reviewer #4 for pointing out the need for improvement in the manuscript’s language. In response, we thoroughly edited the manuscript ourselves and also sought feedback from colleagues fluent in academic English to ensure that the writing is clear, correct, and unambiguous. We believe these revisions have addressed the reviewer’s concern and have strengthened the overall readability and presentation of the manuscript.

**Comment #6:** **Please use the space provided to explain your answers to the questions above. You may also include additional comments for the author, including concerns about dual publication, research ethics, or publication ethics. (Please upload your review as an attachment if it exceeds 20,000 characters)**

Reviewer #4 comment: In methodology, pretested questionnaire is used but validation result is not stated. Study duration not mentioned in methodology part. Clarity on sample size is required as the calculated sample size given is 208 but collected participants number is 139 which needs explanation. In result section, Table 3. The percentage given for categorical variables need to be checked carefully. Little more clarity on explanation is needed for both Table 2 & 3. Continuous variable on Table 2 needs explanation. In figure 1. reported symptom of OADR to AGM was shown to as red eyes be the most followed by stinging and burning sensation, but in results section no red eyes are mention, other than red eyes, it was written as stinging and burning sensation to be the most. It will be good if given the details of ADR produced by individual drug with frequency. In statistical analysis part, only one test has been performed Outcome analysis if performed will impact the study. For overall language, Minor English editing is required.

**Response to Reviewer #4:** We thank the reviewer for the thoughtful and detailed feedback. We have carefully revised the manuscript to address each of the points raised, as outlined below:

i. **Validation of the questionnaire**:

*Reviewer comment*: “Pretested questionnaire is used but validation result is not stated.”

***Response*:** We have now clarified in the *Methods* section that the questionnaire was pre-tested on eight licensed optometrists and subsequently revised for clarity and ease of understanding (see *Methods: Study design, survey tool, setting and population*).

ii. **Study duration**:

*Reviewer comment*: “Study duration not mentioned.”

***Response*:** The study duration (February to May 2024) is found at the beginning of the *Methods* section.

iii. **Sample size vs. actual participants**:

*Reviewer comment*: “Calculated sample size is 208 but collected participants number is 139, which needs explanation.”

***Response*:** We have clarified in the *Methods: Study participants* section that although the calculated sample size was 208, 139 optometrists eventually participated, due to limited availability and response rates. This explanation has been explicitly added to avoid ambiguity.

iv. **Tables 2 and 3 clarity**:

*Reviewer comment*: “Percentages need to be checked; more explanation needed for Tables 2 & 3; continuous variable on Table 2 needs explanation.”

***Response*:** We appreciate the reviewer’s careful attention to detail. The percentages in Tables 2 and 3 were verified and are accurate; however, minor discrepancies occur due to rounding, which has now been clarified in the footnotes. Additional explanatory notes have been added to table 2 to enhance clarity and interpretation. In Table 2, *age* and *practice years* were treated as continuous variables because they represent numerical values measured on a scale with meaningful intervals. Presenting them as mean ± standard deviation (SD) allows for a clearer depiction of the central tendency and variability among participants. This approach provides more nuanced information than categorical grouping, as it enables statistical testing (independent sample *t*-test) to determine whether differences between groups (those who routinely manage glaucoma vs. those who do not, and those reporting ocular ADRs vs. those who do not) are significant. The non-significant p-values in both cases suggest no meaningful difference in mean age or years of practice across the compared groups. Footnote has been added to the table 3 as well.

v. **Figure 1 inconsistency (red eyes vs. stinging/burning sensation)**:

*Reviewer comment*: “Figure 1 shows red eyes as the most reported symptom, but in results section it is stated otherwise.”

***Response*:** We have corrected the inconsistency. The *Results* section has been revised to accurately reflect the data presented in Figure 1.

vi. **Details of ADRs by individual drug**:

*Reviewer comment*: “It will be good if given the details of ADR produced by individual drug with frequency.”

***Response*:** We thank the reviewer for this insightful suggestion. In response, we have provided an additional table 4 that presents the distribution of adverse drug reactions (ADRs) by individual glaucoma medications. This table includes the frequency of each ADR alongside the corresponding medications reported to be associated with them. The inclusion of this table highlights the specific patterns of ADRs attributable to individual drugs and enhances the clarity of our findings.

vii. **Statistical analysis**:

*Reviewer comment*: “Only one test has been performed; outcome analysis would strengthen the study.”

***Response*:** We sincerely appreciate the reviewer’s observation. We acknowledge the importance of outcome analyses in enriching the interpretation of results. However, considering the nature and structure of our dataset, the statistical test we performed was the most appropriate and informative for addressing our study objectives. Additional outcome analyses were carefully considered, but the available data did not permit their meaningful application without risking misinterpretation. We believe the test employed provides the most reliable and valid insights from our dataset, and thus best supports the conclusions drawn.

viii. **Minor English editing**:

*Reviewer comment*: “Minor English editing required.”

***Response*:** As noted previously, we have thoroughly revised the manuscript and sought feedback (language edit) from colleagues in academic Department of English to ensure clarity, readability, and grammatical accuracy.
